# Supplementary material for: Contrast normalisation masks natural expression-related differences and artificially enhances the perceived salience of fear expressions
Source: PLoS One. 2020 Jun 11;15(6):e0234513. doi: 10.1371/journal.pone.0234513 (PMC7289429; doi:10.1371/journal.pone.0234513)
Supplement: S1 Table — Sidak-corrected paired comparisons (α = 0.0127) are performed separately for each frequency category: broadband, low-, mid-, and high-frequency versions of faces. df = 139 for all tests. (DOCX) [file pone.0234513.s001.docx]

| **S1 Table. RMS contrast differences between fear and expression counterparts.** | | | |
| --- | --- | --- | --- |
| Expression comparisons (broadband) | t | Sig | CI |
| **Fear** |  |  |  |
| Neutral | -2.67 | .008 | -.011, -.001 |
| Anger | -5.09 | <.001 | -.016, -.007 |
| Happy | -.19 | .85 | -.005, .004 |
| Disgust | -4.27 | <.001 | -.013, -.004 |
| Expression comparisons (low-frequency) |  |  |  |
| **Fear** |  |  |  |
| Neutral | -.64 | .51 | -.006, -.001 |
| Anger | -2.18 | .03 | -.006, -.001 |
| Happy | -2.55 | .01 | -.008, -.001 |
| Disgust | -.34 | .73 | -.004, .002 |
| Expression comparisons (mid-frequency) |  |  |  |
| **Fear** |  |  |  |
| Neutral | 1.53 | .12 | -3e-4, .003 |
| Anger | 1.82 | .07 | -e-4, .004 |
| Happy | -2.56 | .01 | -.003, -4e-4 |
| Disgust | -5.53 | <.001 | -.009, -.004 |
| Expression comparisons (high-frequency) |  |  |  |
| **Fear** |  |  |  |
| Neutral | -4.49 | <.001 | -.004, -.001 |
| Anger | -9.06 | <.001 | -.012, -.007 |
| Happy | -6.38 | <.001 | -.006, -003 |
| Disgust | -11.77 | <.001 | -.012, -.009 |
| Sidak-corrected paired comparisons (*α*= 0.0127) are performed separately for each frequency category: broadband, low-, mid-, and high-frequency versions of faces. *df*=139 for all tests. | | | |
