# Supplementary material for: Contrast normalisation masks natural expression-related differences and artificially enhances the perceived salience of fear expressions
Source: PLoS One. 2020 Jun 11;15(6):e0234513. doi: 10.1371/journal.pone.0234513 (PMC7289429; doi:10.1371/journal.pone.0234513)
Supplement: S2 Table — Fourier amplitude spectrum between fear expressions and neutral, anger, happy and disgust face counterparts. Paired comparisons are Sidak-corrected paired comparisons (α = 0.0127). df = 139 for all tests. (DOCX) [file pone.0234513.s002.docx]

| **S2 Table. Fourier amplitude spectra.** | | | |
| --- | --- | --- | --- |
| Expression comparisons (Fourier amplitude) | t | Sig | CI |
| **Fear** |  |  |  |
| Neutral | -5.06 | <.001 | -.064, -.028 |
| Anger | -5.31 | <.001 | -.061, -.028 |
| Happy | 2.20 | .02 | .002, .036 |
| Disgust | -1.61 | .10 | -.031, .003 |
| Fourier amplitude spectrum between fear expressions and neutral, anger, happy and disgust face counterparts. Paired comparisons are Sidak-corrected paired comparisons (*α*= 0.0127). *df*=139 for all tests. | | | |
