# Supplementary material for: Contrast normalisation masks natural expression-related differences and artificially enhances the perceived salience of fear expressions
Source: PLoS One. 2020 Jun 11;15(6):e0234513. doi: 10.1371/journal.pone.0234513 (PMC7289429; doi:10.1371/journal.pone.0234513)
Supplement: S3 Table — Sidak-corrected paired comparisons (α = 0.0063) between broadband fear and emotion counterparts. Comparisons in the first instance are calculated using RMS contrast, and Michelson contrast in the second instance. For both sets of comparisons an additional 4 tests were included to identify whether differences are preserved under conditions of manipulation. df = 18 for all comparisons. (DOCX) [file pone.0234513.s003.docx]

| **S3 Table. Apparent contrast comparisons for broadband faces.** | | | |
| --- | --- | --- | --- |
| Apparent contrast (broadband faces) RMS | t | Sig | CI |
| **Fear** |  |  |  |
| Neutral | -3.44 | .003 | -.001, -3e-4 |
| Anger | 1.02 | .32 | -7e-4, .002 |
| Happy | -3.34 | .004 | -.001, -3e-4 |
| Disgust | -3.55 | .002 | -.001, -4e-4 |
| *Manipulated faces* |  |  |  |
| Neutral | -3.51 | .002 | -.002, -6e-4 |
| Anger | -3.49 | .003 | -.001, -4e-4 |
| Happy | -1.27 | .22 | -9e-4, 2e-4 |
| Disgust | -2.26 | .03 | -.002, -1e-4 |
| Apparent contrast (broadband faces) Michelson |  |  |  |
| **Fear** |  |  |  |
| Neutral | 1.91 | .07 | -2e-4, .006 |
| Anger | .71 | .48 | -.002, .004 |
| Happy | .60 | .45 | -.002, .004 |
| Disgust | 2.73 | .01 | .001, .008 |
| *Manipulated faces* |  |  |  |
| Neutral | -.52 | .60 | -.005, .003 |
| Anger | -.003 | .99 | -.003, .003 |
| Happy | 2.02 | .05 | -1e-4, .006 |
| Disgust | 1.64 | .11 | -.001, .009 |
| Sidak-corrected paired comparisons (*α*= 0.0063) between broadband fear and emotion counterparts. Comparisons in the first instance are calculated using RMS contrast, and Michelson contrast in the second instance. For both sets of comparisons an additional 4 tests were included to identify whether differences are preserved under conditions of manipulation. *df*= 18 for all comparisons. | | | |
