# Supplementary material for: Contrast normalisation masks natural expression-related differences and artificially enhances the perceived salience of fear expressions
Source: PLoS One. 2020 Jun 11;15(6):e0234513. doi: 10.1371/journal.pone.0234513 (PMC7289429; doi:10.1371/journal.pone.0234513)
Supplement: S4 Table — Sidak-corrected paired comparisons (α = 0.0063) between low-frequency (LSF) fear expressions and emotion counterparts. Comparisons are calculated using Michelson contrast only; no significant expression effect was observed for the same faces when RMS contrast was the metric. df = 18 for all comparisons. (DOCX) [file pone.0234513.s004.docx]

| **S4 Table. Apparent contrast comparisons for LSF faces.** | | | |
| --- | --- | --- | --- |
| Apparent contrast (LSF faces) Michelson | t | Sig | CI |
| **Fear** |  |  |  |
| Neutral | .17 | .86 | -.006, .007 |
| Anger | -1.33 | .19 | -.010, .002 |
| Happy | .91 | .37 | -.003, .008 |
| Disgust | 1.40 | .17 | -.001, .009 |
| *Manipulated faces* |  |  |  |
| Neutral | -.02 | .98 | -.006, .006 |
| Anger | -1.46 | .16 | -.013, .002 |
| Happy | .02 | .98 | -.006, .006 |
| Disgust | .79 | .43 | -.004, .010 |
| Sidak-corrected paired comparisons (*α*= 0.0063) between low-frequency (LSF) fear expressions and emotion counterparts. Comparisons are calculated using Michelson contrast only; no significant expression effect was observed for the same faces when RMS contrast was the metric. *df*= 18 for all comparisons. | | | |
