# Supplementary material for: Contrast normalisation masks natural expression-related differences and artificially enhances the perceived salience of fear expressions
Source: PLoS One. 2020 Jun 11;15(6):e0234513. doi: 10.1371/journal.pone.0234513 (PMC7289429; doi:10.1371/journal.pone.0234513)
Supplement: S5 Table — Sidak-corrected paired comparisons (α = 0.0063) between mid-range frequency (MSF) fear expressions and emotion counterparts. Comparisons in the first instance are calculated using RMS contrast, and Michelson contrast in the second instance. For both sets of comparisons an additional 4 tests were included to identify whether differences are preserved under conditions of manipulation. df = 18 for all comparisons. (DOCX) [file pone.0234513.s005.docx]

| **S5 Table. Apparent contrast for MSF faces.** | | | |
| --- | --- | --- | --- |
| Apparent contrast (MSF faces) RMS | t | Sig | CI |
| **Fear** |  |  |  |
| Neutral | -1.25 | .22 | -.7e-4, 1e-4 |
| Anger | -.71 | .48 | -6e-4, 3e-4 |
| Happy | -2.79 | .01 | -.001, -1e-4 |
| Disgust | -6.01 | <.001 | -.001, -8e-4 |
| *Manipulated faces* |  |  |  |
| Neutral | -1.30 | .20 | -8e-4, 1e-4 |
| Anger | -1.77 | .09 | -.001, 1e-4 |
| Happy | -1.60 | .12 | -8e-4, 1e-4 |
| Disgust | -5.65 | <.001 | -.001, -6e-4 |
| Apparent contrast (MSF faces) Michelson |  |  |  |
| **Fear** |  |  |  |
| Neutral | -3.28 | .004 | -.015, -.003 |
| Anger | -4.78 | <.001 | -.021, -.008 |
| Happy | -2.79 | .01 | -.018, -.002 |
| Disgust | -2.42 | .02 | -.011, -8e-4 |
| *Manipulated faces* |  |  |  |
| Neutral | -3.08 | .006 | -.016, -.003 |
| Anger | -5.21 | <.001 | -.029, -.012 |
| Happy | -1.60 | .12 | -.011, .001 |
| Disgust | -.57 | .57 | -.006, .003 |
| Sidak-corrected paired comparisons (*α*= 0.0063) between mid-range frequency (MSF) fear expressions and emotion counterparts. Comparisons in the first instance are calculated using RMS contrast, and Michelson contrast in the second instance. For both sets of comparisons an additional 4 tests were included to identify whether differences are preserved under conditions of manipulation. *df*= 18 for all comparisons. | | | |
