# Supplementary material for: Contrast normalisation masks natural expression-related differences and artificially enhances the perceived salience of fear expressions
Source: PLoS One. 2020 Jun 11;15(6):e0234513. doi: 10.1371/journal.pone.0234513 (PMC7289429; doi:10.1371/journal.pone.0234513)
Supplement: Attachment — Submitted filename: response_letter_final.docx [file pone.0234513.s007.docx]

Dear Editor and Reviewers,

We would like to thank you for your comments and suggestions for the attached manuscript, and the time that it has taken to provide these. We have found these to be very helpful, and feel that given these changes, the manuscript has benefitted from additional clarity and improved presentation. All suggested changes have been made, and below (green text) are responses that address each point. Please note that we have also updated the format in some areas of the document so that these are consistent with Plos One requirements. All changes have been recorded using tracked changes.

We look forward to hearing about the progress of the revised manuscript.

Thank you again,

Regards,

Abigail Webb

**Comments from Reviewer 1. Responses in green text.**

- The authors indicated that the participants all "had normal or corrected to normal vision". Did the authors actually measure their participants' visual acuity, or did they just assume that it was "normal"? If the authors measured acuity, please report average acuity for the group (and range of measured acuities).

  We did not measure visual acuity. The study advert advised participants to wear glasses if they would normally do so when using a computer. This has now been clarified.
- Procedure, lines 164-166. The authors say that the first session collected data from 19 participants. They then say that "data collection was added for an additional 19 participants". Does this mean that there were a total of 38 participants? If so, this conflicts with the previous specification (on line 138) that "nineteen individuals took part in the study". These statements are confusing and apparently contradictory; please clarify.

Thank you for prompting this clarification. There were indeed *overall* 38 participants. Initially our intention was to include 3 conditions for broad, low and high frequency facial expressions. This represented the first round of data collection, and was intended initially to be the only round of data collection -19 participants were tested here. We decided to include a condition for mid-range frequency faces, simply to include a middle ground between low and high frequency cutoffs. To achieve this we tested an additional 19 participants, who were presented with mid-range filtered faces only. This has now been clarified in the text.

- The manuscript says that the experimental data are "under embargo" and includes a "private link". My understanding is that the published article in PLOS ONE should provide readers with access to all relevant data. While access to the data is clearly provided to the reviewers, I am expecting the authors to make the data available publicly as well. Please confirm.

  Apologies for the confusion here. The submitted manuscript included a private link to an embargoed dataset. The intention here was to provide data for reviewers, and to change this to a publicly-available status in the event of manuscript submission. On Figshare, I have now updated the license (C0) and the data is now public. DOI for this is included in the manuscript ([10.6084/m9.figshare.9944216](https://doi.org/10.6084/m9.figshare.9944216))
- With regards to Figure 1 and the associated pairwise comparisons, I will trust the authors' reports of significant differences between the contrasts associated with fear and the other emotions, but the differences look so small. What are the numerical differences? Are these differences in contrast (while statistically significant), large enough to be practically important? Can the authors plot these results in a way that the obtained differences are more perceptually visible to readers? What about plots of contrast DIFFERENCE between fear and the other emotions (instead of absolute magnitudes, as shown in Figure 1). As things stand now, the reported differences don't look compelling (in both Figures 1 and 2). It is really hard to see meaningful differences in the slopes of the curves (a significant difference in slope was reported in the manuscript between fear and the other expressions) shown in Figure 2.

To elucidate the size/meaningfulness of expression differences in apparent contrast we have now included associated percentage increase/decreases in cases where Sidak-corrected comparisons were statistically significant.

As both reviewers have both highlighted the hard-to-see differences in Figure 1, we have now included a log axis so that these differences are more apparent (though please note that Figure 1 now appears as Figure 2 in the revised manuscript). Changes also include replacement of STDVs with SEMs. Thank you also for suggesting clarification for Figure 2 (Now Figure 3 in revised manuscript). Alteration of axes means that information contained in each face as spatial frequency increases is now clearer between expressions.

- With regards to the matching task, from the figures provided, it is not readily evident from the plots that fear is all that different from the other expressions, despite the reported statistical outcomes. Are the small statistical differences found in this study between fear and the other expressions truly of a magnitude that creates practical and meaningful effects? I trust that this is so, but these are my thoughts. The authors' conclusions sound important, but the differences between fear and the other expressions usually appear so small in the manner that they are plotted now.

Thank you for highlighting this issue. To visually clarify the size of differences between expressions, Figures for contrast matching data (now Figs 4-5 in revised manuscript) now include a log y axis, and SEMs have replaced STDVs. In terms of quantifying these differences meaningfully, the revised manuscript also includes percentage increase/decreases for statistically significant comparisons.

- The error bars shown in Figure 1 are not described in the figure caption (standard deviations?, standard errors?)

  The error bars shown in Figure 5 are not described in the figure caption (standard deviations?)

  All figure error bars now depict SEMs as opposed to STDs. The caption for Figure 6 in the revised manuscript includes a notation to highlight that some error bars are smaller than marker sizes.

**Comments from Reviewer 2. Responses in green text.**

- L.112. It is not fully clear how or in which way the present study extends previous findings. Authors should be more explicit. For instance, I would invite the authors to openly state what is the specific advancement of this work compared to previous research.

  Some clarifications and elaborations have been made in the revised manuscript, and the final paragraph of the introduction now reads:

  The objective of the present study is to extend our understanding of the mechanisms that drive biases for fearful face expressions, in order to establish the importance of image contrast in the threat bias for fearful faces. The first question that we addressed was whether there are naturally occurring differences in contrast across expressions, prior to contrast normalisation, which could affect the salience of these stimuli. Our first experiment therefore quantifies naturally occurring differences in physical contrast across expressions, and how this differs across different spatial scales. The second question that we addressed was whether there were would be differences in the apparent contrast of expressions. It has been suggested that fearful expressions might be more salient because their Fourier amplitude spectra well matched to the contrast sensitivity function of the human visual system. If so, then this would be expected to result in an increase in their apparent contrast, which in turn might then be the causal explanation of the threat bias. Our second experiment therefore measures expression-related differences in apparent, perceived contrast between facial expressions. Together, these results allow us to quantify the extent to which the threat bias can be explained in terms of naturally occurring differences in physical and effective contrast.
- L.121 and L.136 :
  It would be clearer to call this subsection "experiment 1" and then "Experiment 2" such as to make it consistent with what is said in L.112 and L.114. And present each section as a separate experiment.

  There are now subheadings for Methods and Results sections denoting each experiment. I believe that this clarifies the structure of the manuscript, thank you for this suggestion.
- L133. Can the authors please make the MATLAB code used for both features available? otherwise specify specifically the steps taken to measure both features.

  The following text has been added to the methods to provide clarification:

1. Each image was cropped to a 300x200 region including the internal features of the face.
2. Pixel intensity values were scaled so that the minimum possible value (black) was zero and the maximum possible value (white) was1.
3. RMS contrast was calculated as standard deviation of the scaled pixels intensities.
4. Fourier transform was calculated using the MATLAB fft2() function, and the amplitude of each pixel in the transformation using the MATLAB abs() function.
5. For each image, pixels were binned according to their spatial frequency (across all orientations) using 100 bins, and taking the mean value of all pixels in each bin. The mean across all images for each expressions was calculated, and plotted in Fig 3.
6. For each image, the Fourier amplitude slope was estimated by performing a linear regression of log(spatial frequency) against log (amplitude).

- I beliebe that the correct 1997 reference by E.Peli (#26) to which the authors refer to is "Pelli D. G. (1997). The VideoToolbox software for visual psychophysics: Transforming numbers into movies. Spatial Vision, 10, 437–442." And not the one titled "In search of a contrast metric...". Is this possible?

Yes. Thank you for pointing out this error. Reference number has now been corrected and replaced with Pelli (1997), and its reference number adjusted.

- L.161 Could the authors please add visuals to illustrate the procedure timelines?

An additional figure has been added as a task schematic. This is now Figure 1, and all other Figure numbers have been adjusted accordingly.

- L164. Did the trials include both instances of faces (normal and manipulated?). If so, please specify here (again) too.

Yes. Manipulated control faces were intermixed with normal, upright faces. Clarification has now been included within the manuscript text.

- L165.Can the authors please justify in the text why they tested the MSF faces separately?.

  Thank you to both reviewers for prompting this clarification. As noted in the response to Reviewer 1: There were indeed *overall* 38 participants. Initially our intention was to include 3 conditions for broad, low (<1 cpd) and high frequency (>6 cpd) facial expressions. This represented the first round of data collection, and was intended initially to be the only round of data collection -19 participants were tested here. We decided to include a condition for mid-range frequency faces (1<6cpd), to ensure a middle ground between low and high frequency cutoffs. This is important given that, in face perception literature, labels for frequency cutoffs and their associated values vary between studies. “Low” has referred to <0.8 and <0.2 cpd, and “high” to >3.3, >6, and >8cpd. To make sure we addressed this “range” of cutoffs used in other studies, our frequency cutoffs included low (<1cpd), mid-range (1<6cpd) and high (>6cpd). We therefore tested an additional 19 participants, who were presented with mid-range filtered faces only. This has now been clarified in the text.

  Please see the following articles for examples of variable frequency cutoffs:

  - **Low (<0.8 cpd) high (>3.3 cpd)**Bannerman, R. L., Hibbard, P. B., Chalmers, K., & Sahraie, A. (2012). Saccadic latency is modulated by emotional content of spatially filtered face stimuli. *Emotion*, *12*(6), 1384.
  - **Low (<2 cpd) high (>6 cpd)** Stein, T., Seymour, K., Hebart, M. N., & Sterzer, P. (2014). Rapid fear detection relies on high spatial frequencies. *Psychological science*, *25*(2), 566-574.
  - **Low (<2cpd) high (>8cpd)** Holmes, A., Green, S., & Vuilleumier, P. (2005). The involvement of distinct visual channels in rapid attention towards fearful facial expressions. *Cognition & Emotion*, *19*(6), 899-922.
- L.174. It is a bit unclear why it is stated in this title that the results are those between broadband AND spacially filtered expressions but later (L178) it is specified that the ANOVA is for each spacial frequency separately. This should be consistent.

The title for this subsection has now been changed- there is no longer a suggestion of differences *between* frequency categories, rather the new title suggests they are treated separately. “Experiment 1: RMS contrast for broadband and spatially filtered expressions”.

- L.177. Please check again the bar graphs of Figure 1. They do not seem correct. They all look flat. Overall, I would advice that the authors use the SEM in their error bars everywhere.

  Y axes Figure 2 have now been updated to a more appropriate log scale, which also clarifies the differences (please note that the addition of a task schematic means that Figure 1 is now Figure 2 in the revised manuscript). All error bars in bar plots now refer to SEM, and captions have been adjusted accordingly.
- Could the authors please specify in the text on what are the ANOVAs being performed?. (e.g., "Repeated measures ANOVA was performed on..... for each spatial frequency". It will help the reader.

  Please note that at the beginning of each results subsection we state the kind of ANOVA that is conducted.
- Please check the reporting style. equality symbols are constantly missing throughout the manuscript.

  Thank you for highlighting the missing equality symbols. These are now present in the revised manuscript.
- The authors first talk about two experiments..which actually point out to two stages of their procedure. They later present results in one major section, instead of making explicit that there are two results section and finally introduce an "experiment 3", which is actually the results of post-hoc analyses which are actually subsequent analyses added to further understand the results of the "experiment 2"...
  I think that the authors must clarify the parts in the manuscripts' methods and results section and be consistent with what they present. Perhaps a structure in "Experiment 1" and then "Experiment 2" totally separate could bring clarity.

  Thank you for this suggestion, based on a previous comment from Reviewer 2 (prompting clearly defined subheadings for Experiments 1-3), new subsections in Methods and Results in the revised manuscript now clearly state the experiment number. There is now clearer separation throughout the manuscript.
- L.384. I believe that the discussion section is lacking some development relative to the emotion-emotion comparisons, either in the RMS analyses or the contrast matching. I wonder if the authors can elaborate more on these. I think this is key to understand the mechanisms by which fear may or may not have the saliency effects and crucially bring more insight into the "adaptive pressure" vs. "low-level" tenets to which they refer in the introduction. For instance, it seems that in many of their results (e.g., the pairwise comparisons) that happy facial expressions are somehow immune to contrast differences compared to fearful faces. But also the rest of the comparisons are also important to mention. This could be implemented in a paragraph in the discussion.

  Thank you to both reviewers for highlighting room for clarification in the discussion. Some clarifications and elaborations have been made in the revised manuscript, and the first paragraph of the discussion has been expanded to summarise the results and their implications:

  The present study extends the current understanding of the way in which facial expressions differ in terms of their physical composition, and importantly, how such differences are affected by spatial filtering techniques. Recent findings show that facial stimuli differ compared to images of natural scenes in terms of their Fourier amplitude spectra, and that differences also exist *within* face categories, where fear expressions tend to be lower in RMS contrast compared to other expressions [23, 30]. Our findings support the notion that facial expressions are associated with naturally-occurring differences, both at the physical and subjective level. This is true in terms of the physical contrast and Fourier amplitude spectra (Experiment 1), and the way in which expressions differ in terms of their perceived salience, measured by apparent contrast (Experiment 2). Moreover, the present studies extend this understanding, confirming that the RMS contrast of fearful faces tends to be lower than that of other expressions, but also that this tendency for lower RMS contrast for fear expressions becomes increasingly apparent as faces are filtered to contain only high spatial frequencies. These findings have important consequences for studies of facial expressions that employ spatial filtering techniques to explore “low road” approaches to the threat bias for fear expressions. Findings show that combinations of spatial filtering and contrast normalisation will tend to alter the relative contrast of stimuli compared to that found under natural viewing, where these effects disproportionately alter the physical and perceived appearance between expressions of different emotions.

- L.418 what do the error bars represent in Fig1?.

  All figure error bars now represent standard errors, and captions have been corrected to reflect this.
